# AGE MODERATES THE ASSOCIATION BETWEEN IMPLICIT AND EXPLICIT NEGATIVE EMOTIONAL REACTIVITY

**DOI:** 10.1093/geroni/igz038.1120

**Published:** 2019-11-08

**Authors:** Mai B Mikkelsen, Gitte Tramm, Mimi Mehlsen

**Affiliations:** Department of Psychology and Behavioural Sciences, Aarhus University, Aarhus, Denmark

## Abstract

Background: According to the notion of maturational dualism, the link between mind and body weakens with age and this weakening has important consequences for emotional experiences. Specifically, it is hypothesized that age-related decline in interoceptive awareness and physiological reactivity reduce the ability to use bodily states to guide judgments about emotions. If this hypothesis is valid, then age may moderate the association between explicit measures (based on consciously accessible mental representations of affect) and implicit measures (based on unconscious affective processes such physiological activation) of affective reactivity. Purpose: To investigate whether age moderates the association between explicit and implicit measures of negative affective reactivity. Methods: A sample of 275 participants (age range=20-78) viewed 25 pictures validated to induce negative emotions. Participants filled in the PANAS assessing explicit affect and the IPANAT assessing implicit affect before and after viewing the pictures. Emotional reactivity was operationalized as residualized gain scores derived from regressions of baseline affect on affect following picture viewing. Results: Age moderated the association between implicit negative affective reactivity and explicit negative affective reactivity (B=60). Discussion: The results showed a reduced association between explicit and implicit negative affective reactivity with age. This finding is consistent with the notion of maturational dualism and may indicate that older adults use affective processes that are not represented consciously (e.g., physiological activation) less than young adults when judging their emotional state.

